# Mental health and psychiatric care in Bolivia: what do we know?

**DOI:** 10.1186/1752-4458-8-18

**Published:** 2014-05-15

**Authors:** Denisse Jaen-Varas, Wagner Silva Ribeiro, Jessie Whitfield, Jair de Jesus Mari

**Affiliations:** 1 Departamento de Psiquitria, Universidade Federal de São Paulo, Rua Borges Lagoa, 570, São Paulo, SP 04038-000, Brasil; 2 Saint Louis University School of Medicine, St. Louis, Missouri, USA

**Keywords:** Mental health service, Public policy, Health policy, Developing countries, Bolivia, South America, Alcohol Addiction, Domestic Violence, Suicide

## Abstract

**Background:**

Recently Bolivia has implemented a universal health system, but their mental health policy is still emerging.

**Objectives:**

To investigate the current state of the mental health care system in Bolivia and discuss challenges for structuring a coordinated network of services that can effectively meet the needs of the Bolivian population.

**Methods:**

This review was conducted by searching for scholarly articles through the databases Lilacs, Medline OPS, HISA and IBECS REPIDISCA via the search portal in the Virtual Health Library - NLM (http://www.bireme.br).

**Results:**

Bolivia has a National Mental Health Plan that is intended to guide mental health promotion, prevention, treatment and rehabilitation of mental illness, but the resources for this area of health are limited. There are 1.06 psychiatrists and 0.46 psychologists per 100, 000 inhabitants. Information on psychiatric morbidity in Bolivia and the impact of mental disorders on the global burden of disease is scarce. Admission statistics reported by psychiatric hospitals in the country show that the main cause of hospitalization is substance abuse (30%). Alcohol consumption is responsible for 90% of these admissions, in addition to being a major cause of deaths in traffic and one of the main risk factors for domestic violence. Almost one in two women in Bolivia (47%) experienced some form of violence from their partner in the last year. Nineteen percent of women living with a partner reported being physically abused, while 7% were sexually abused by their partners. Isolated studies report that suicide rates are disproportionately high in Bolivia.

**Conclusions:**

Although there is a shortage of epidemiological data in Bolivia, it is clear the impact of alcohol addiction in psychiatric admissions, domestic violence and traffic accidents. Violence against women and suicides are important issues to be tackled. Among the proposed strategies to afford human resources for mental health in Bolivia, “task shifting”, the delegation of tasks to non-specialists should be extensively adopted in the country to improve mental health care.

## Background

Bolivia is located in Latin America and has 10,027,254 inhabitants, according to the 2012 Census [1]. The Bolivian population is characterized by a diversity of ethnicities and cultures, with more than 36 ethnic groups identified [2]. The most representative ethnic groups are the Quechua (30%), Aymara (25%) Chiquitano (2.2%) and Guarani (1.5%) [2], while 58% of the Bolivian population descend from a combination of indigenous groups and Spanish colonizers from the 1500s [1,2]. A third of the population still lives in rural areas [3-5]. With a gross domestic product (GDP) of approximately U.S. $ 27 billion in 2012, Bolivia is considered a lower middle income country according to World Bank’s criteria [6]. According to the United Nations (UN), Bolivia ranked 108^th^ in the 2012 Human Development ranking with a Human Development Index (HDI) of 0.675, which was lower than the regional average for Latin America and the Caribbean (0.741) and the world’s average (0.694) [7]. Bolivia also holds the 14^th^ position in the ranking of economic inequality, with a Gini of 53.0 [8]; the percentage of people living in poverty is 64%, and the illiteracy rate is estimated at 14% [9].

Worldwide, life expectancy at birth is 64 years for men and 67 years for women, but Bolivia ranks last in life expectancy among the eight countries of South America [9]. A healthy life expectancy in Bolivia is up to 54 years [9]. Of every 1,000 live births, 46 infants die before reaching one year of age, and 54 die by the fifth year of life [3,7]. While 35% of the deaths in Bolivia are still attributed to infectious diseases, noncommunicable diseases are responsible for 57% of the mortality rate [10]. Of the eight countries in South America, Bolivia is sixth for population of all ages living with HIV, with an average of 17,000 HIV positive people [11].

Based on the measurement of years lived with disability (YLDs), the five leading causes of burden of disease in Bolivia are: iron deficiency anemia, back pain, major depressive disorder, neck pain, and anxiety disorders [12]. Although communicable diseases remain the leading causes of disability-adjusted life years (DALYs), their contribution to the burden of disease has decreased since 1990. Conversely, the percentage of DALYs attributable to noncommunicable diseases has been increasing since then [12].

Unlike other Latin American countries [13], Bolivia, until recently, did not have a universal health care system. As a result, more than two thirds of the population had no access to medical care [14]. With regard to mental health services, the limited information available in the literature suggests the absence of well-structured care network for the treatment of mental disorders [15,16]. However, this information is not enough for us to have a clear picture of the current state of policies and the network of mental health care in Bolivia.

The main objective of this paper is to investigate the current status of the mental health system in Bolivia by comparing the guidelines and standards established by the legislation to the actual availability of resources for the treatment of people with mental illness. This paper also aims to delineate the progress in the implementation of Bolivia’s mental health policy and to discuss the challenges for structuring a coordinated network of services that can effectively meet the need of the Bolivian population.

## Review

### Methods

This article is based on a review of the scientific literature about systems and services for mental health care in Bolivia. This review was conducted by searching for scholarly articles through the databases Lilacs, Medline OPS, HISA and IBECS REPIDISCA via the search portal in the Virtual Health Library - NLM (http://www.bireme.br). Articles published in Spanish and English up to 09/22/2013 were included. Also consulted were references of selected articles and newsletters of the World Health Organization (WHO) and Pan American Health Organization (PAHO). We also made contact with Bolivian researchers in an attempt to find unpublished articles, theses and other materials.

To study the mental health policy in Bolivia and the status of its mental health care network, the official documents of the database of the Ministry of Health and Sports of Bolivia were sought (http://www.sns.gob.bo). To obtain additional information, or to access documents that, although mentioned on the Ministry’s webpage and other sites, were not included in the database, personal contacts were made and correspondence (physical and electronics) conducted with the Ministry. The contact details of these representatives were obtained at the website of the Ministry.

### Results

Bolivia has had a universal health care system only since 2007, when the New Political Constitution of the State (*Nueva Constitución Politica del Estado –* NCPE) was promulgated [17]. Since then, all Bolivians have had a constitutional right to health, which must be provided by the state free of charge, and without discrimination of any kind [17]. But according to the Ministry, 77% of the population has been excluded from health services due to economic, geographic, cultural and social barriers [14]. The NCPE, along with the Sector Development Plan, prioritizes health promotion and disease prevention, which also includes traditional medicine as an alternative treatment, retrieving the knowledge and practices [17,18].

The System of National Health is organized into three levels of care. According to 2007 data from the National System on Health Information (*Sistema Nacional de Información en Salud – SNIS*), there were 3,145 primary health care centers: 2,875 first-level, 210 second-level and 60 tertiary-level centers; within the 1st level of care, 45% of the establishments did not have a physician [15,19].

The first institution for the treatment of mental disorders in Bolivia was the National Institute of Psychiatry Gregorio Pacheco in the city of Sucre, which was founded in 1884 by then President Gregorio Pacheco [20-22]. For many decades, the hospital functioned primarily as a “haven for patients suffering from mental disorders”. Since 1959 the Hospital Order of St. John of God took over the institution. In 1986, there was a major restructuring and expansion of the National Institute of Psychiatry, where new units were built and expanded [20]. Despite the changes, 307 out of 369 beds in the hospital were intended for medium and long hospital stay [20]. Currently the hospital is divided into three units: acute, intermediate, and chronic, the latter serving to accommodate patients that are impaired and in neglectful social or family situations. The same applies to the child psychiatric unit that houses patients up to 18 years who are impaired or without family support; these patients are directed to chronic unit.

Since 1999 there have been initiatives to develop programs for mental health [19], including the Action Plan for Mental Health [23]. In 2000, Bolivia was one of five countries in South America that had developed a mental health plan (Bolivia, Chile, Colombia, Uruguay and Venezuela) [24]. According to information from the World Health Organization, this plan was not implemented due to lack of financial support [15]. There are few laws or decrees in mainstream health policy that fall under the purview of mental health. Among them, there are laws targeting the reduction of substance use, the care for victims of natural disasters, the prevention of domestic violence and the protection of human rights for people living with HIV/AIDS [19,25]. In order to cope with the lack of regulations, mental health professionals, via their professional associations, have developed policies for the prevention of mental disorders and the promotion of mental health [15]. In the department of La Paz (2005), an instrument was designed to address the integration of mental health into the primary care network. It consisted of: 1) organization of services and staff training; 2) monitoring, 3) definition of procedures for care and 4) community participation [23].

In 2009, the National Mental Health Plan 2009–2013 was launched as a response to the lack of public policies on mental health [19]. This plan is intended to guide actions to promote mental health, support the prevention, treatment and rehabilitation of mental illness, and specialize efforts to benefit the most vulnerable populations: children and adolescents of both sexes, women of reproductive age, peasant indigenous populations and the elderly [19]. The main axes of the Mental Health Plan are:

1. Promotion of mental health as inseparable part of comprehensive health and human development, with the aim to implement strategies and guidelines for promoting mental health and preventing mental illness through an ongoing educational process;

2. Development and implementation of primary health care standards and procedures to facilitate an approach to mental health service networks that ensures the human rights of patients and employs 1st, 2nd and 3rd care levels in coordination with one another;

3. Coordination with other players and sectors involved with strategic mental health promotion to strengthen mental health networks and the prevention of mental disorders. One objective here is to request support from institutions and public-private partnerships for implementation and achievement of successful advocacy and prevention.

With the help of the Assessment Instrument for Mental Health Systems of the World Health Organization (WHO-AIMS), information was collected in 2008 on Bolivia’s mental health system, the results are as follow [15]:

WHO estimates for 2008 indicate that, in quantitative terms, Bolivia earmarked 0.2% of its health budget for mental health [15], meaning that about 600,000 Bs (about $ 75,000 per year) was directed towards the program of National Mental Health, Prevention and Rehabilitation [16];

Per 100,000 population, Bolivia has 1.06 psychiatrists, 0.34 nurses, 0.46 psychologists, 0.25 social workers, 0.20 occupational therapists and 1.43 for other health professionals [15];

There is little mental health training for primary care health personnel, and only an estimated 2% of medical training is devoted to mental health. There are few patients and family associations that receive support from the government [15].

In 2008 Bolivia had 39 outpatient mental health centers throughout the country. Only one interfaced directly with the community, yet there was no mobile team of mental health professionals. All mental health services for outpatient clinics had at least one psychotropic medication. Bolivia also had 12 day-treatment facilities (seven of which were for children and adolescents) and 9 psychiatric hospitals throughout the country (giving a rate of 9.56 psychiatric beds per 100,000 inhabitants). In the country there are no mental health services for incarcerated people with mental disorders [15]. The percentage of women attending the outpatient service is higher (54%) than men, but in other services this relationship is reversed, especially for day treatment (where 33% attendees are women). The length of stay in psychiatric hospitals is twice (74 days per year) that of time spent in inpatient units (35 days per year, specifically in psychiatric units and wards at the General Hospital) [15].

There is little data on the prevalence of mental disorders in Bolivia or their impact on the global burden of disease. Admission statistics reported by psychiatric hospitals in the country show that the main cause of hospitalization is substance abuse (30%). Alcohol consumption is responsible for 90% of these admissions, in addition to being a major cause of deaths in traffic and one of the main risk factors for domestic violence [16]. Psychotic disorders are the second leading cause of hospitalization with 29%, and mood disorders ranked fourth with 13.5% [16], although depression is the most common general disorder and the tenth most frequent complaint seen by any general doctor in Bolivia [2]. An important problem in several countries of the region is the underreporting of suicide mortality [26]. Isolated studies report that rates are disproportionately high in Bolivia; suicide deaths may cause up to 40% of youth mortality in the city of Alto (located in the city of La Paz), while in the community rates have been reported as high as 430 per 100,000 population [16]. Currently we do not have a database with statistics for mental health care in Bolivia.

Available data [27,28] show that one in two women in Bolivia (47%) experienced some form of violence from their partner in the last year. Nineteen percent of women living with a partner reported being physically abused, while 7% were sexually abused by their partners. Most victims of sexual abuse were also victims of physical or psychological abuse. Moreover, one in five Bolivian women (21%) reported that they suffered psychological abuse by their partners, although they were not physically or sexually abused. The authors conclude that intimate partner violence is common in Bolivia [27,28]. Comparative results of mental health indicators between Bolivia and other countries in South America can be found in Table 1.

**Table 1:** A comparison of social and demographic factors between South America countries

	**BOLIVIA**	**BRAZIL**	**ARGENTINA**	**CHILE**	**ECUADOR**	**PARAGUAY**	**PERÚ**	**URUGUAY**
Population (Total) (2012)	10,027,254	198,656,019	41,086,927	17,464,814	15,492,264	6,687,361	29,987,800	3,395,253
Rural population (% of total population) (2012)	33	15	7	11	32	38	22	7
GDP (Current US$) (2012)	27,035,110,130	2,252,664,120,777	470,532,788,510	268,187,780,226	84,039,856,000	25,502,060,502	196,961,048,689	49,059,705,180
GINI	53,0	51.9	45.8	52.1	47.7	53.2	46.0	45.3
IDH (2013)	0,675	0.730	0.811	0.819	0.724	0.669	0.741	0.792
Population in poverty % (2011)	64,0	22.0	33.8	16.9	25.0	Rural 43.4	52.0	32.1
						Urbana 39.8		
Life expectancy at birth (years) (2011)	M 64	M 70	M 72	M 76	M 73	M 70	M 72	M 73
	F 67	F 77	F 80	F 82	F 79	F 74	F 77	F 80
Incidence of tuberculosis (per 100,000 people) (2012)	131	42	26	18	62	45	101	21
Maternal mortality ratio (modeled estimate, per 100,000 live births) (2010)	190	56	77	25	110	99	67	29
Mortality rate, infant (per 1,000 live births) (2012)	46	13	13	8	20	19	14	6
Mortality rate, under-5 (per 1,000 live births) (2012)	54	14	14	9	23	22	18	7
Prevalence of HIV, total (% of population ages 15-49) (2012)	0.3	0.5	0.4	0.4	0.6	0.3	0.4	0.7
Budget allocated to mental health of the total health budget (2011)	0.2	2.35	2.0	2.14	1.2	1.0	3.0	7.0
Psychiatric beds available per 100,000 (2011)	9.6	27.2	17	9.1	12	7.8	4.0	34.9
Rates of psychiatrists per 100,000 (2011)	1.06	3.20	9.20	4.70	2.00	1.31	2.10	19.36
Rates of psychologists per 100,000 (2011)	0.46	10.19	106	12.30	1.34	28.94	1.78	3.12
Rates of nurses per 100,000 (2011)	0.34	1.69	NA	1.70	0.94	1.58	1.94	0.69

Sources: NA = Not applicable.

## Conclusion

This article attempts to provide an overview of mental health policy and the structure of mental health services in Bolivia. While Bolivia has in the past been one of the first countries in South America to have drafted a mental health plan, which never became effective in practice due to the lack of financial funding [16]. Since 2009, the National Mental Health Plan, together with the New State Constitution, ensures that health is a right for all Bolivians. Additionally, laws were developed and regulatory issues explored that indirectly promoted mental health. Bolivia lacks epidemiological data that can guide the formulation of policies. Available data, however, suggests that alcohol consumption is the leading cause of hospitalization, and that suicide is an important issue, at least among specific populations, such as youth leaving in certain areas of the country. There is also evidence that some of the major risk factors for common mental disorders, such as intimate partner violence, are common in Bolivia.

Information on psychiatric morbidity in Bolivia and on the impact of mental disorders on public health is limited. Currently the international literature indicates that mental and behavioral disorders are among the leading causes of burden of disease worldwide (13%) [29]. In addition, mental disorders tend to persist throughout the lifecourse, causing significant long-lasting impairment [26]. The literature emphasizes the need for further investment in mental health care systems. Despite current efforts to enhance the mental health care system in Bolivia, the country still invests very little in mental health, with only 0.2% of the total budget for health care allocated specifically for mental health care [15]. This is much less than the average budget for mental health care in South America, which is estimated to be 2.36% [9]. Bolivia still lacks epidemiological data based on which mental health policy should be established. Therefore, the production of such data might be an important tool to engage policymakers in the restructuring of the mental health system.

When discussing a new era of mental health policies in Bolivia, the National Mental Health Plan 2009–2013 should be mentioned. This plan was adopted in Bolivia in 2009 and supports the promotion of mental health and the prevention of mental illnesses, as well as treatment and rehabilitation of people with mental disorders. In addition, it recommends the coordination of mental health care with primary care and other sectors that are involved with issues relating to mental health. This plan is in accordance with international conventions and modern psychiatry, as well as with the best practices of community-based services. It also aims to ensure that those with mental illness receive a humane and integrated care, and that, in cases that require hospital admission, the inpatient treatment should be short-stay to facilitate their reintegration into society as soon as possible. Although the Mental Health Plan is very well designed, the resources devoted to mental health are still insufficient.

Despite the progress initiated by the National Mental Health Plan, many issues relating to mental health need to be addressed. These include: an insufficient amount of services, lack of qualified professionals, poor mental health training for primary care staff, and extended stays in psychiatric hospitals, among others. A comparison with a neighbouring country in South America such as Brazil, which ranks sixth in GDP in the world, shows that Brazil also has a small number of psychiatrists (3.2 psychiatrists per 100,000 population) [9,13,30]. It should be noted that the number of psychiatrists in both Bolivia (1.6 psychiatrists per 100,000) and Brazil is below what the WHO recommends, which is 5–9 psychiatrists per 100,000 inhabitants [30]. In order to cope with the needs for mental health care, Brazil has implemented Psychosocial Community Centers (CAPS), which are distributed through the entire country (current mental health policy establishes that there should be a CAPS for each 200,000 inhabitants in the country). Besides being responsible for treating severe mental disorders, CAPS’ multidisciplinary mental health teams provide guidance, training and supervision to primary care health workers, who are responsible for treating mild and moderate mental disorders. In addition, such teams perform several cultural and educational activities within the community, which aim at reducing stigma, preventing mental health problems and integrating patients into the social context. Such model might be adopted and implemented in Bolivia as a strategy for providing mental health care for those in need.

Given the challenges and limited resources, some alternatives for structuring a network of mental health care for the Bolivian population include the following:

Task shifting [31-33]: there has been proposed that non-specialist health professionals, such as general practitioners, nurses, social workers, community health workers, etc., can provide safe and effective treatment for mental illnesses, as long as they are properly trained and supported by mental health specialists. In Bolivia, mental health professionals should be responsible for providing training, supervision and support to traditional practitioners and community representatives, as well as to medical doctors working at the Program for Intercultural Family Health Care in the Community (Salud Familiar Comunitaria Intercultural - SAFCI). As the SAFCI is Bolivia’s main strategy to provide primary care throughout the country, integrating mental health care into this program should be a priority in order to guarantee access to care for all people with mental illnesses. Such model is in line with current human-rights principles, which state that all individuals with mental illnesses should be offered equal access to services that are located within the community, and which should safeguard their autonomy and self-determination [34].

In addition, research needs to be conducted on the effectiveness of treatments that are culturally appropriate and acceptable. As traditional medicine is deeply integrated into Bolivia’s cultural context, it is vital to take into account the beliefs, traditions and culture of patients with mental health problems. Traditional practitioners might play an important role in the treatment, identification and referral of mental health patients.

The infants and adolescents require special attention for the prevention and treatment of mental illness, due to the treatment gap in childhood and adolescence is even greater than in adults [35]. Most mental illnesses in childhood involve development processes, so reducing the duration at this stage of the disease, and concentrating resources at this early stage, could revolutionize treatment [29].

Finally, an integration between different sectors, such as practitioners, researchers and policymakers should be promoted in Bolivia, so the country should effectively structure and implement a mental health policy which meets the needs of the Bolivian population.

## Competing interests

The authors declare that they have no competing interests.

## Authors’ contributions

DJV, WSR and JJM conceived the article; DJV carried out the review and prepared the manuscript; WSR and JJM supervised the review and the manuscript preparation; DJV, WSR, JW and JJM contributed to the discussion, conclusions and final revision of the article. All authors read and approved the final manuscript.
